# Short-course PD-1 blockade in locally advanced nasopharyngeal carcinoma: a phase II randomized trial protocol (Tori-013)

**DOI:** 10.3389/fimmu.2025.1633243

**Published:** 2025-08-18

**Authors:** Hongyuan Jia, Junchao Wang, Ling Zhang, Haijun Li, Yalei Du, Wenjuan Luo, Zou Wei, Xiaohui Wang, Weidong Wang

**Affiliations:** ^1^ Department of Radiation Oncology, Sichuan Clinical Research Center for Cancer, Sichuan Cancer Hospital and Institute, Sichuan Cancer Center, University of Electronic Science and Technology of China, Chengdu, China; ^2^ Department of Oncology, The General Hospital of Western Theater Command, Chengdu, China; ^3^ Department of Oncology, The Second People’s Hospital of Neijiang, Neijiang, China; ^4^ Department of Oncology, The Second People’s Hospital of Yibin, Yibin, China; ^5^ Department of Oncology, Mianyang 404 Hospital, Mianyang, China; ^6^ Department of Radiation Oncology, Meishan Cancer Hospital, Meishan, China; ^7^ Department of Oncology, Sichuan Friendship Hospital, Chengdu, China

**Keywords:** nasopharyngeal carcinoma, toripalimab, PD-1 inhibitor, chemoradiotherapy, efficacy and safety

## Abstract

While immunotherapy has demonstrated encouraging efficacy in locally advanced nasopharyngeal carcinoma (LANPC), the optimal combination modalities and treatment duration remain undetermined. In the present study, we developed a clinical trial protocol to evaluate shortened period of immunotherapy could enhance the efficacy of LANPC. This open-label, randomized, single-blind, multicenter phase II trial (Tori-013) investigates the efficacy and safety of toripalimab (anti-PD-1 monoclonal antibody) combined with induction chemotherapy (IC) followed by concurrent chemoradiotherapy (CCRT) in patients with stage III/IVa nasopharyngeal carcinoma (NPC). Eligible participants (estimated n=154) are randomized 1:1 to receive either IC (gemcitabine + cisplatin) plus CCRT (cisplatin + radiotherapy ≥ 70 Gy) with toripalimab (240 mg, Q3W) or placebo. Toripalimab/placebo is administered during IC and CCRT phases, followed by two additional cycles post-radiotherapy. The primary endpoint is 3-year progression-free survival (PFS), with secondary endpoints including overall survival (OS), objective response rate (ORR), Epstein-Barr virus (EBV) DNA dynamics, lymphocyte subset changes, and safety. Safety assessments focus on immune-related adverse events (irAEs) graded by CTCAE v5.0. Approved by the Ethics Committee of Sichuan Cancer Hospital (KY-2021-113) and registered (ChiCTR2200055494), this trial aims to establish a novel, streamlined immunochemoradiotherapy strategy for locally advanced NPC, potentially enhancing efficacy while maintaining tolerability

## Introduction

Nasopharyngeal carcinoma (NPC) is a malignant tumor originating from the epithelial cells of the nasopharynx, most commonly arising in the fossa of Rosenmüller. It is distinct from other head and neck cancers due to its unique geographic distribution, strong association with Epstein–Barr virus (EBV) infection, and high sensitivity to radiotherapy (1). In 2022, an estimated 120,416 individuals worldwide were newly diagnosed with nasopharyngeal cancer, with high incidence in certain regions, particularly in Southern China, Southeast Asia and North Africa (2).

Over 70% of NPC cases are initially diagnosed as local advanced nasopharyngeal carcinoma (LANPC) (3). The foundational treatment for this stage of diseases is concurrent chemoradiotherapy (4). In recent years, multiple large-scale randomized clinical trials have demonstrated that the combination of induction chemotherapy with concurrent chemoradiotherapy (IC+CCRT) result in high tumor regression rates and promising long-term survival outcomes (5). As a result, current clinical guidelines endorse IC+CCRT as a standard therapeutic approach for managing locoregionally advanced NPC (6, 7). Among various induction regime, gemcitabine plus cisplatin has been shown with good efficacy and well tolerated. However, in the era of IC+CCRT there are still some patients suffered from recurrent or metastatic diseases, thus new treatment modality is needed to ameliorate the efficacy of LANPC.

Immunotherapy offers compelling biological advantages in the treatment of NPC, owing to its distinct etiological and immunological characteristics. Non-keratinizing NPC is etiologically linked to EBV infection, and the expression of EBV-encoded latent proteins and viral antigens contributes to the tumor’s inherent immunogenicity, thereby providing a strong rationale for immunotherapeutic interventions (8, 9). Moreover, elevated expression of programmed death-ligand 1 (PD-L1) is frequently observed within the NPC tumor microenvironment, particularly on infiltrating immune cells, rendering immune checkpoint blockade a biologically plausible and clinically actionable strategy (10). The tumor immune milieu of NPC is also characterized by a high density of tumor-infiltrating lymphocytes (TILs), especially cytotoxic CD8^+^ T cells, further underscoring the potential for immune activation and therapeutic responsiveness (11). These biological features establish a strong foundation for the use of immune checkpoint inhibitors in NPC, as demonstrated in clinical trials. Many random clinical trials were designed to evaluate various combinations of chemoradiotherapy with different PD-1 inhibitors. Possible combinations include addition of PD-1 inhibitor as adjuvant immunotherapy; ‘sandwich type’, namely addition of PD-1 inhibitor in induction plus adjuvant immunotherapy (13); whole course immunotherapy, for which PD-1 inhibitor is administrated throughout induction, concurrent and adjuvant phase. While some results among them have been reported and demonstrated promising efficacy, the best combination modality of PD-1 inhibitor in LANPC is still unknow.

Toripalimab is a humanized IgG4κ monoclonal antibody targeting PD-1, which have been approved in China in November 2021 as a first-line treatment for recurrent or metastatic nasopharyngeal carcinoma based on the phase 3 JUPITER-02 trial. This trial demonstrated its efficacy in combination with gemcitabine and cisplatin compared to placebo plus chemotherapy (12, 14). In our study, we aim to demonstrate addition of toripalimab to induction chemotherapy and concurrent chemoradiotherapy is safe and could improve the outcomes of LANPC. The efficacy and safety of this combination mode have not been reported in previous randomized trials.

## Methods

### Study design

The study is a single blinded, multi-center, randomized, phase II clinical trial designed to evaluate the efficacy and safety of toripalimab in combination with induction chemotherapy followed by CCRT and additional two cycles of immunotherapy in patients with locally advanced NPC. To minimize confounding factors, a stratified block randomization approach will be implemented. Patients will be stratified by age (≤60 *vs*. >60 years), sex (male *vs*. female), AJCC stage (III *vs*. IVa). Patients are blinded to treatment allocation, while investigators are unblinded for safety management and regimen adjustment. For control of bias, independent Endpoint Review Committee (IERC) assesses radiographic endpoints in blinded fashion. The Data and Safety Monitoring Board (DSMB) composed of medical oncologists, radiation oncologists, immunologists, and biostatisticians. The DSMB will review safety data, including the incidence and severity of adverse events (AEs), especially immune - related adverse events (irAEs), at regular intervals during the trial have authority to recommend trial modification/termination based on predefined safety stopping rules. The Ethics Committee of Sichuan Cancer Hospital approved this study (Ethics Approval Number: KY-2021-113). The trial was registered with the Chinese Clinical Trial Registry (Registration Number: ChiCTR2200055494). The study began across sixteen medical centers from January 2023.

### Inclusion criteria

Patients with stage III–IVa NPC, as defined by the American Joint Committee on Cancer (AJCC) 8th edition staging system, were eligible for inclusion. Patients were eligible if they met the following criteria: histologically confirmed diagnosis of locally advanced NPC (stage III–IVa); age 18–70 years; Karnofsky performance status (KPS) ≥ 70; no prior history of immunotherapy, and no active autoimmune disease; adequate organ function.

### Exclusion criteria

Exclusion criteria included: prior head and neck cancer treatment (surgery, chemotherapy, or radiotherapy); active infection or severe systemic illness; pregnancy or breastfeeding. The main inclusion and exclusion criteria are listed in **Table 1**.

**Table 1:** Key eligibility criteria of the study.

Inclusion criteria	Exclusion criteria
Age 18–70 years, any gender.	Prior treatment with PD-1, PD-L1, PD-L2, CTLA-4 antibodies, or other immune checkpoint inhibitors.
Pathologically confirmed NPC (Stage III – IVa, T1-2N2-3/T3-4N0-3M0 per AJCC 8th edition).	Participation in other drug trials within the past month.
At least one measurable lesion per RECIST 1.1 via CT or MRI.	Known severe allergy to monoclonal antibodies or components of study drugs.
No prior anti-cancer treatment.	History of severe allergic reactions or atopic constitution.
No contraindications to radiotherapy, chemotherapy, or immunotherapy.	Known history of other malignancies.
Availability of tumor tissue for PD-L1 testing (new biopsy preferred or archived FFPE tissue).	≥ Grade 2 peripheral neuropathy (NCI CTCAE v5.0).
Karnofsky Performance Status ≥ 70.	Interstitial lung disease or non-infectious pneumonitis (excluding local radiation-induced).
Adequate hematologic function (HGB ≥ 90 g/L, WBC ≥ 4×10^9^/L, PLT ≥ 90×10^9^/L).	Uncontrolled systemic diseases (e.g., diabetes, hypertension, acute pulmonary conditions).
Adequate liver function (ALT/AST <1.5×ULN, TBIL <1.5×ULN).	Active infection requiring systemic therapy, including TB.
Adequate renal function (serum creatinine <1.5×ULN; endogenous creatinine clearance rate ≥ 60 mL/min).	Significant cardiovascular disease (e.g., NYHA class ≥ II, recent MI, arrhythmia).
Normal coagulation (INR/PT ≤1.5×ULN).	Active HBV or HCV infection (e.g., HBV DNA ≥ 10³ copies/L; HCV RNA positive).
Normal cardiac biomarkers and thyroid function.	Immunodeficiency or history of organ transplant, HIV positive.
	Ongoing systemic corticosteroid or immunosuppressive therapy; active autoimmune disease.
	Major surgery within 4 weeks prior to Day 1 or unhealed wounds/fractures.
	Live vaccine within 4 weeks prior to study drug (except inactivated flu/COVID-19 vaccines).
	Antibiotic therapy within 4 weeks prior to first dose.
	Drug abuse or psychiatric disorders interfering with compliance.
	Pregnancy, breastfeeding, or planning pregnancy during study and within 180 days after.

NPC, nasopharyngeal carcinoma; CT, computed tomography; MRI, magnetic resonance imaging; RECIST, Response Evaluation Criteria in Solid Tumors; ULN, upper limit of normal; UILN, upper international limit of normal; ALT, alanine transaminase; AST, aspartate transaminase; HGB, hemoglobulin; WBC, white blood cell; PLT, platelet; TB, Tuberculosis; FFPE, Formalin-fixed paraffin-embedded.

### Screening

The screening and baseline assessments will be conducted within 0–14 days prior to enrollment to confirm eligibility and establish baseline parameters. Informed consent must be obtained prior to any study-related procedures. Medical history, including height and weight will be recorded, and participants will be evaluated against inclusion/exclusion criteria. Tumor tissue samples will be collected for PD-L1 expression analysis via immunohistochemistry. Pre-treatment blood samples will also be centrally stored. Imaging assessments include nasopharynx and neck MRI, chest CT, abdominal ultrasound, and whole-body bone scintigraphy (ECT). Alternatively, 18F-FDG PET/CT may replace chest CT, abdominal ultrasound, and ECT if performed. Laboratory evaluations encompass EBV DNA quantification, complete blood count, biochemistry etc. All data and samples will be managed per protocol to ensure consistency across the multicenter study.

### Intervention

**Figure 1:**
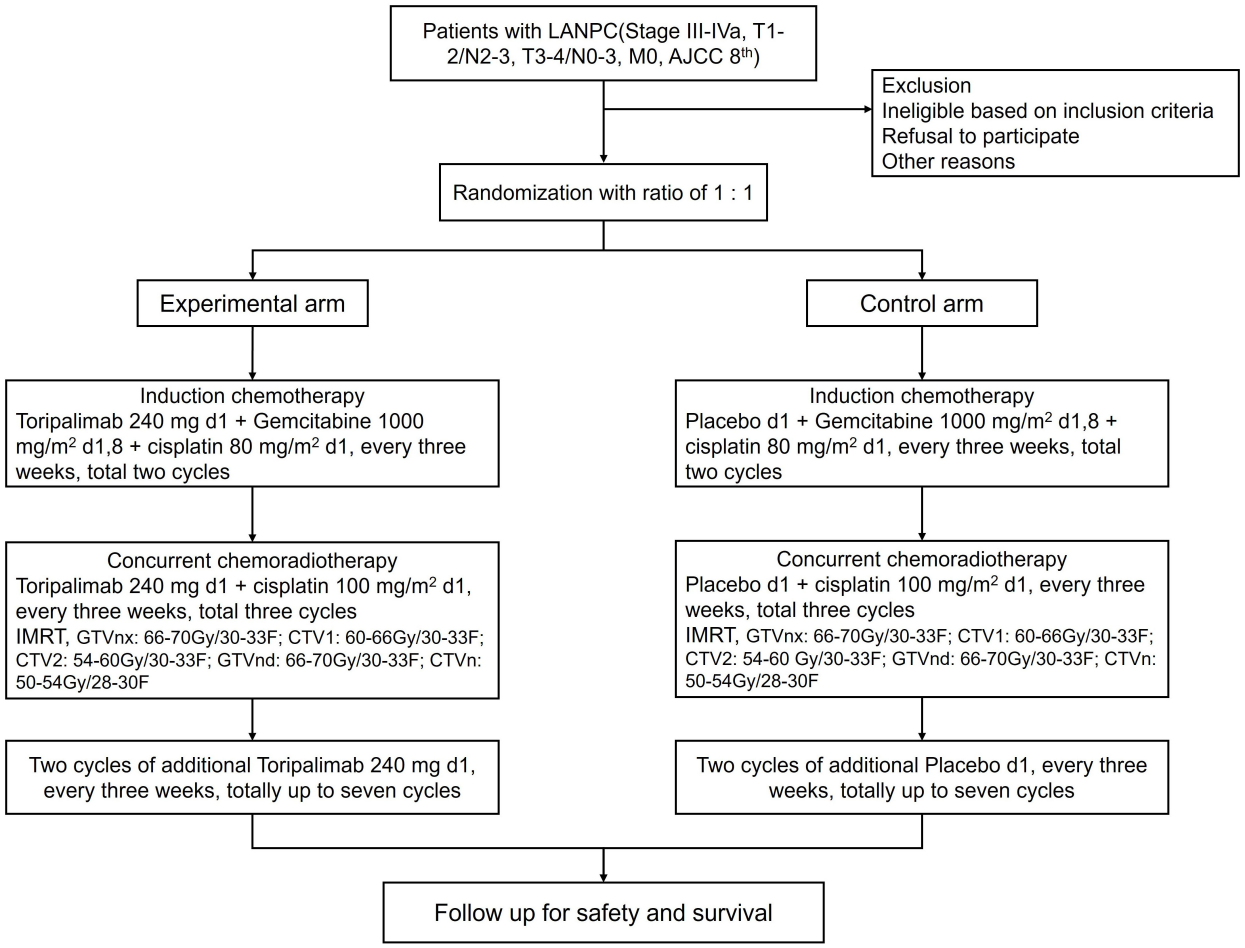
Flowchart of the study.

Patients were randomly assigned to the two treatment arms in a 1:1 ratio, using stratified permuted block randomization with the aforementioned stratification factors: CCRT followed by induction chemotherapy plus placebo (control arm) and CCRT followed by induction chemotherapy plus toripalimab (experimental arm). Regime of induction chemotherapy is gemcitabine (1200 mg/m^2^ d1, 8) plus cisplatin (75 mg/m^2^ d1).CCRT consists of cisplatin-based chemotherapy (100 mg/m² every 3 weeks for 3 cycles) and radiotherapy (≥ total dose of 70 Gy delivered over 7 weeks). Toripalimab (240 mg, intravenous) or placebo is delivered every 3 weeks starting from induction chemotherapy. After the end of chemoradiotherapy, additional two cycles of toripalimab or placebo are administrated up to a total of seven cycles. The flow chart of this study is illustrated in **Figure 1**.

### Follow-up

Patients will undergo tumor assessments at baseline (screening phase) and every 12 weeks (± 7 days) during the first year post-treatment initiation. Subsequent follow-up evaluations will occur every 3 months for the first 2 years, every 6 months from years 3 to 5, and annually thereafter. Data collection and management will be performed using electronic case report forms (eCRFs). Follow-up evaluations will include contrast-enhanced MRI scans of the nasopharynx and neck, chest CT, abdominal ultrasound, nasopharyngoscopy, and laboratory testing of EBV DNA and lymphocyte subsets in peripheral blood. Survival status, subsequent therapies, and quality of life (QoL) will be documented at each visit. Key safety assessments (e.g., blood counts, biochemistry) and imaging schedules align with protocol-defined intervals. Detailed timing of enrollment, interventions, and evaluations is summarized in **Table 2**.

**Table 2:** Flowchart of enrollment, interventions, and assessments.

Study phase	Screening	Treatment visit IC cycle 1	Treatment visit IC cycle 2	Treatment visit CCRT cycle 3	Treatment visit CCRT cycle 4	Treatment visit CCRT cycle 5	Treatment visit IO cycle 6 - 7	Follow-up (every 3–6 months)
Timepoint	-d14 - d0	d1 (w1d1)	d22 (w4d1)	d43 (w7d1)	d64 (w10d1)	d85 (w13d1)	d106 (w16d1), d127 (w19d1)	
Visit No.	1	2	3	4	5	6	7	8
Informed Consent	X							
Inclusion/Exclusion Criteria	X							
Medical History	X							
Physical examination	X	X	X	X	X	X	X	X
Vital Signs	X	X	X	X	X	X	X	X
KPS	X	X	X	X	X	X	X	X
Concomitant Medications	X	X	X	X	X	X	X	X
Head MRI	X		X			X		X
Chest CT	X							X
Abdominal US	X							X
ECT	X							
ECG	X	X	X	X	X	X	X	X
Echocardiography	X							
Laboratory Tests (CBC)	X	X	X	X	X	X	X	X
EBV DNA	X		X			X		X
Biopsy tissue sample	X							
Lymphocyte subset test	X		X			X		
Thyroid function	X							X
Serum autoantibodies	X							X
Nasopharyngeal endoscope	X							X
Adverse Event/SAE Recording	X	X	X	X	X	X	X	X
QoL-Questionnaires	X		X			X		X
Survival status							X	X
Review of subsequent therapy							X	X

CT, computed tomography; MRI, magnetic resonance imaging; US, ultrasound; EBV, Epstein-Barr virus; KPS, Karnofsky performance status, IO, immunotherapy; IC, induction chemotherapy; CCRT, concurrent chemoradiotherapy; ECG, electrocardiograph; ECT, emission computed tomography; QoL-Questionnaires, quality of life-questionnaires; SAE, sever adverse event.

### Outcome measures

The primary endpoint was progression-free survival (PFS) at 3 years, defined as the time from randomization to disease progression (as assessed by RECIST 1.1) or death from any cause. The secondary endpoints included: Overall survival (OS): Defined as the time from randomization to death from any cause. Objective Response Rate (ORR): Defined as the proportion of patients with a complete response (CR) or partial response (PR) as assessed by RECIST 1.1 criteria. Biological markers include copy number of EBV and change of lymphocyte subsets of periphery blood. To reveal deeper mechanistic insight, functional immune profiling will be performed on pretreatment and subsequent serial biopsies collected when possible during the study, including tumor-infiltrating immune cell phenotyping, T-cell exhaustion markers (e.g. PD-1, TIM-3, LAG-3), cytokine production, etc. The process of enrollment, interventions, and assessments is summarized in **Table 2**.

### Safety assessment

Safety was assessed based on the incidence of adverse events (AEs) according to the Common Terminology Criteria for Adverse Events (CTCAE) version 5.0. The frequency and severity of adverse events were categorized as grade 1–5, with special attention to irAEs such as pneumonitis, hepatitis, and colitis. Given that nasopharyngeal carcinoma is often associated with EBV infection, there is a possibility that EBV-related immune dysregulation may contribute to the development of atypical immune - mediated toxicities during immunotherapy, such as immune - mediated hepatitis. To address this concern, we implemented several procedures. We will monitor EBV DNA copy number every 2 cycles. Baseline serum autoantibodies (e.g. ANA, anti-dsDNA, anti-thyroglobulin) will be assessed before treatment and re-assessed when indicated to rule out active autoimmune diseases. For patients who develop ≥ Grade 3 irAEs, inflammatory cytokine signatures will be tested, including interleukin-6 (IL-6), interferon-gamma (IFN-γ), and tumor necrosis factor-alpha (TNF-α). We grade and manage irAEs according to Following NCCN guidelines. Generally, for Grade 1 irAEs, close observation and continuation with immunotherapy. For Grade 2 irAEs, temporarily withhold immunotherapy until downgrading and prescribe specific medications. For Grade 3–4 irAEs, permanently discontinue immunotherapy, use high-dose IV corticosteroids with tapering, and promptly offer intensive care for life - threatening cases. If the incidence of grade 3–4 irAEs in either treatment arm exceeds 30% or any life - threatening irAEs occur in more than 5% of patients in either arm during the study, the DSMB will immediately review the cases and may recommend halting the study to re-evaluate the safety profile of the treatment.

### Sample size calculation

According to data reported in the literature, the 3-year PFS rate for the induction chemotherapy followed by chemoradiotherapy group is approximately 80% (5). The expected 3-year PFS rate for the toripalimab plus induction chemotherapy followed by chemoradiotherapy group is 90%. With an enrollment period of 24 months, a follow-up duration of 36 months, and an annualized dropout rate of 5%, this study plans to randomize 154 subjects (77 in each arm). The study aims to achieve 90% nominal statistical power to demonstrate superiority of the experimental arm over the control arm at a one-sided significance level of 0.025.

### Statistical analysis

The Full Analysis Set (FAS) includes all randomized subjects who received at least one drug infusion, excluding only those disqualified during the run-in period or lacking post-enrollment follow-up. The Per Protocol Set (PPS), a subset of FAS, comprises subjects with valid baseline data, full protocol compliance, and treatment adherence (80%–120%). The Safety Analysis Set (SS) covers all randomized subjects who received ≥ 1 drug infusion and post-treatment safety evaluations. Continuous variables (e.g., laboratory values) are summarized as mean ± standard deviation. Within-group pre-post changes are analyzed with paired t-tests; between-group differences are assessed via ANOVA adjusted for center effects. Categorical variables (e.g., adverse event rates) are described using frequencies and compared via Chi-square tests, including total and AE-related dropout rates. AE rates are compared using Chi-square tests. Survival endpoints (e.g., PFS) are analyzed via Kaplan-Meier curves and log-rank tests. As a pre-specified sensitivity analysis, a Cox proportional hazards model will be implemented to estimate hazard ratios (HR) only if ≥30 progression/death events are observed (ensuring ≥10 events per covariable), adjusting for potential confounding factors. ORR comparisons will utilize Chi-square tests, with exact logistic regression incorporated if covariate imbalances emerge post-randomization. All analyses were conducted using SPSS software. A two-sided test will be conducted for all statistical tests, and a p-value of < 0.05 will indicate that the differences are significant.

### Data collection, management, and monitoring

Data will be collected electronically via secure, password-protected case report forms (eCRFs), with source documents (imaging reports, lab results) uploaded and cross-verified centrally. All participants will be assigned unique coded identifiers, and their personal identifying information will be anonymized during the study. Investigators are responsible for completing CRFs for each enrolled subject. Prior to site initiation or data entry, investigators and authorized staff will receive appropriate training. Only authorized study personnel, who have completed training on data privacy and security protocols, will have access to this database. Encrypted CRFs will be reviewed by the clinical monitor and promptly transferred to the data manager for data entry and management. Data Monitoring Committee (DMC) will review eCRFs to assess completeness and consistency, cross-checking critical data against source documents. Missing or inconsistent data identified during monitoring or via electronic data capture (EDC) system validation checks will trigger electronic queries; site investigators must resolve these within a predefined timeframe through source verification, data correction/provision, or documented justification within the EDC system (maintaining audit trails). Unresolved queries will be escalated to the Principal Investigator (PI) and central management team. All data recording, corrections, and modifications are the responsibility of the investigator or designated personnel. The final database will be locked only after resolution of all outstanding queries.

## Discussion

In LANPC, the combination of immunotherapy with chemoradiotherapy has become a focal point of current research. Previous studies have explored various integration strategies—such as adjuvant immunotherapy, whole-course immunotherapy, and the “sandwich” approach—with encouraging outcomes. However, to date, no randomized controlled trial results have been published regarding the use of immunotherapy during both the induction and concurrent chemoradiotherapy phases. To the best of our knowledge, the present study is the first random controlled trial to evaluate the efficacy and safety of the addition of toripalimab in induction and concurrent phase in patients with LANPC.

The efficacy of immunotherapy in LANPC has been partially tested by previous studies. In CONTINUUM study, 425 patients with LANPC were randomly assigned in a ratio of 1:1 to received whole course sintilimab or placebo up to 12 cycles comprising induction, concurrent and adjuvant phase. After median follow-up of 41.9 months, event-free survival at 3 years in sintilimab group (86% [95% CI, 81–90]) was significantly higher than placebo group (76% [95% CI, 70–81]) (15). The phase 3 multicenter DIPPER trial demonstrated that adjuvant camrelizumab significantly improved 3-year event-free survival (EFS) in patients with LANPC following induction and concurrent chemoradiotherapy. The treatment was generally well tolerated, with manageable adverse effects and no notable decline in quality of life (16). In a phase II randomized controlled trial, Liu et al. investigated the efficacy of the “sandwich” approach. The study enrolled 150 patients with stage III–IVa nasopharyngeal carcinoma with baseline EBV DNA levels exceeding 1500 copies/mL. Participants were randomized in a 2:1 ratio to receive two cycles of neoadjuvant chemotherapy combined with toripalimab or placebo, followed by concurrent chemoradiotherapy and then eight cycles of adjuvant immunotherapy or placebo. The 2-year PFS in the experimental arm demonstrated a significant improvement with 92.0% (95% CI, 86.7–97.3) versus 74.0% (95% CI, 61.8–86.2), respectively. There were no significant differences in acute or late adverse events between the two groups, indicating that the addition of immunotherapy did not increase safety risks (13). A single-arm phase II trial demonstrated that, following induction chemotherapy combined with immunotherapy, subsequent treatment with radiotherapy plus immunotherapy yielded a 3-year failure-free survival (FFS) rate of 88.5% (95% CI, 83.4–93.8) and a 3-year OS rate of 97.9%, with a favorable toxicity profile (17). This de-intensified immunoradiotherapy strategy is currently under investigation in an ongoing phase III clinical trial (18). A summary of these trials is presented in **Table 3**. Compared to previous research, the current study employs a distinct combination modality of immunotherapy with IC+CCRT. With only 7 total cycles of immunotherapy administered—the lowest number among comparable studies—the addition of immunotherapy primarily concentrates on the induction and concurrent chemoradiotherapy phases. Following radiotherapy completion, patients merely require two additional standalone immunotherapy sessions. This optimized approach not only enhances treatment accessibility but also significantly reduces both logistical challenges and financial burdens for patients.

**Table 3:** Trials of immune checkpoint inhibitors (ICIs) in combination with chemoradiotherapy in LANPC.

Trial number	Number	Drug	Combination modality	Results	Primary endpoint	Inclusion criteria	Phase	Refs
NCT04907370	494	Toripalimab	induction; ICIs + radiotherapy *vs* chemoradiotherapy	Ongoing	PFS	T4N1/T1-4N2-3	III	(18)
NCT03984357	152	Nivolumab	induction; ICIs + radiotherapy	completed	PFS	T4N1M0 or T1-4N2-3M0	II	(17)
NCT03925090	150	Toripalimab	induction and adjuvant phase: ICIs *vs* placebo	completed	PFS	III-IVa	II	(13)
NCT03700476	425	Sintilimab	whole course: induction, concurrent and adjuvant phase	completed	EFS	III–IVa locoregionally advanced nasopharyngeal carcinoma (excluding T3–4N0 and T3N1)	III	(15)
NCT03427827	450	Camrelizumab	adjuvant ICIs *vs* placebo	completed	EFS	T4N1M0 or T1-4N2-3M0	III	(16)
NCT04446663	50	Toripalimab	IC+CCRT+ICIs *vs* parallel control	Not posted	Safety	T3-4N0-1M0/T1-4N2-3M0	IIa	(19, 20)
NCT03619824	40	Sintilimab	IC+CCRT+ICIs	Not posted	Safety	III-IVa	II	(21, 22)

ICIs, immune checkpoint inhibitors; IC, induction chemotherapy; CCRT, concurrent chemoradiotherapy; RT, radiotherapy; PFS, progression-free survival; EFS, event-free survival; LANPC, locally advanced nasopharyngeal carcinoma; EBV, Epstein-Barr virus; TNM, Tumor/Node/Metastasis staging system (AJCC 8th edition).

There are two studies (NCT04446663 and NCT03619824) with a research design similar to ours in which immunotherapy is administrated in induction and concurrent phase. The NCT04446663 trial is a parallel controlled phase IIa clinical study with a planned enrollment of 50 patients. The experimental group underwent three cycles of albumin-bound paclitaxel + cisplatin induction chemotherapy combined with toripalimab or placebo, followed by concurrent chemoradiotherapy with placebo. Usage of toripalimab is up to six cycles (19, 20). NCT03619824 is a single-arm phase II study designed to evaluate the efficacy and safety of sintilimab (an anti-PD-1 antibody) in combination with induction chemotherapy (gemcitabine + cisplatin) followed by concurrent chemoradiotherapy. Patients receive three cycles of gemcitabine+cisplatin (GP)-based induction therapy with sintilimab, followed by concurrent chemoradiotherapy and maintenance immunotherapy for up to six cycles (21, 22). The trial aims to enroll 40 participants with locally advanced disease. Compared to our study, these trials have a primary endpoint focused on safety, a smaller sample size and a non-placebo controlled design. The results of these studies have not yet been published.

Immunotherapy in EBV-associated nasopharyngeal carcinoma warrants specific vigilance for atypical immune-mediated toxicities. While irAEs are well-characterized with PD-1 inhibitors, their profile may be influenced by underlying EBV-driven immune dysregulation (23). Activation of latent EBV or dysregulated antiviral immune responses during checkpoint blockade could potentially contribute to aberrant inflammation and tissue damage, including immune-mediated hepatitis. This may occur through mechanisms such as epitope spreading, bystander T-cell activation, or amplification of pre-existing autoimmune tendencies in susceptible hosts (24). To address these potential interactions, our protocol incorporates several measures including enhanced EBV DNA monitoring, baseline autoimmunity risk stratification, and standard management of irAEs, as specified in the Method section.

While this trial addresses an important clinical question, several limitations warrant consideration. Firstly, as a phase II study powered primarily for PFS rather than OS, the sample size may limit the statistical power to detect differences in secondary endpoints such as OS or rare adverse events. Secondly, the patient-blinded (investigator-unblinded) design may introduce performance bias, particularly in subjective endpoint assessments like quality of life. However, this is mitigated by independent blinded review of primary radiographic endpoints. Third, the trial is conducted in a multicenter Chinese population with high EBV prevalence. Results may not be fully generalizable to other ethnic groups or regions with lower EBV infection rates. In the future, a larger phase III validation involving multiple ethnic groups would be needed to establish definitive clinical benefit.

In summary, this study is a randomized controlled phase II clinical trial designed to evaluate the efficacy and safety of a novel treatment protocol for locally advanced nasopharyngeal carcinoma, which integrates immunotherapy during both induction and concurrent chemoradiotherapy phases, followed by only two additional cycles of immunotherapy post-radiotherapy. This novel combination strategy of immunotherapy with chemoradiotherapy has not been previously reported in randomized controlled studies, potentially offering a more streamlined therapeutic approach while maintaining clinical effectiveness.
